# Feasibility of the Italian version of the Teen Online Problem Solving Program (I-TOPS) in a sample of adolescents with acquired brain injury: Results from a randomized controlled trial

**DOI:** 10.1371/journal.pone.0354280

**Published:** 2026-07-28

**Authors:** Claudia Corti, Marta Papini, Sandra Strazzer, Renato Borgatti, Romina Romaniello, Geraldina Poggi, Fabio A. Storm, Cosimo Urgesi, Ashok Jansari, Shari L. Wade, Alessandra Bardoni

**Affiliations:** 1 Acquired Brain Injury Unit, Scientific Institute, IRCCS E. Medea, Bosisio Parini, Lecco, Italy; 2 Neuro-oncological and Neuropsychological Rehabilitation Unit, Scientific Institute, IRCCS E. Medea, Bosisio Parini, Lecco, Italy; 3 Neuropsychiatry and Neurorehabilitation Unit, Scientific Institute, IRCCS E. Medea, Bosisio Parini, Lecco, Italy; 4 Bioengineering Lab, Scientific Institute, IRCCS E. Medea, Bosisio Parini, Lecco, Italy; 5 Laboratorio di Neuropsicologia dello Sviluppo, Scientific Institute, IRCCS E. Medea, Pasian di Prato, Udine, Italy; 6 Department of Psychology, Goldsmiths, University of London, London, United Kingdom; 7 Department of Pediatrics, University of Cincinnati College of Medicine, Cincinnati, Ohio, United States of America; 8 Division of Pediatric Rehabilitation Medicine, Cincinnati Children’s Hospital Medical Centre, Cincinnati, Ohio, United States of America; National Trauma Research Institute, AUSTRALIA

## Abstract

Numerous neurocognitive and behavioral deficits in adolescents with acquired brain injury have been associated with impairments in executive functions. Nevertheless, many adolescents do not receive targeted rehabilitation. One of the most validated interventions addressing executive dysfunction in this population is the web-based Teen Online Problem Solving (TOPS) program, developed in the United States in the early 2000s. This study aimed to evaluate the feasibility of the Italian version of TOPS (I-TOPS) in its first implementation in Italy, compared with an active control wellness intervention in adolescents aged 11–19 years. Data on the feasibility of both the training and the study design and procedures are reported from the randomized controlled trial conducted to assess feasibility prior to a full evaluation of efficacy. Forty-two adolescents participated, 21 allocated to I-TOPS and 21 to the wellness intervention. Overall willingness to participate in the study, assessed before randomization across the full sample, was moderate (61.73%). The I-TOPS group, but not the wellness group, demonstrated adequate training adherence, training satisfaction and participation rates. No technological issues hindering program completion, accessibility difficulties with the web-based platform or significant loss to follow-up among participants who completed the training were reported in either group. However, challenges in collecting outcome measures on parent functioning due to refusal were observed in both groups. Adherence to the planned assessment timeline was satisfactory but not fully met. In sum, the findings suggest that I-TOPS represents a feasible approach that is subjectively perceived as useful and engaging by adolescents with acquired brain injury and their families. Nevertheless, the moderate acceptance rate in the study and the potential stigma associated with reporting mental health concerns among some parents may limit the generalizability of the findings. These factors should be taken into account when planning efficacy studies. This study was registered at ClinicalTrials.gov (NCT05169788).

## Introduction

Acquired brain injuries (ABI), either of traumatic (traumatic brain injury-TBI) or non-traumatic origin (non-TBI), are known to cause important long-term cognitive sequelae, with cascade effects on academic and vocational attainment, emotional and behavioral regulation and social interaction [1–7]. The National Center for Health Statistics in the USA estimated that in 2022 2.3 million (3.20%) children and adolescents aged ≤ 17 years received a diagnosis of a concussion or brain injury in the country [8]. An epidemiological study focused on TBI only and based on data collected across North America, Europe, Australia and New Zealand indicated that, each year, approximately 691/100.000 children receive emergency care, with 74/100.000 requiring hospitalization and 9/100.000 resulting in fatal outcomes [9]. Each year in England and Wales, approximately 1.40 million individuals visit emergency departments as a result of head injuries. Of these cases, between one-third and one-half involve children under the age of 15 [10]. Although epidemiological rates vary significantly across countries and studies, the picture reported above provides a clear indication of the magnitude of the phenomenon.

The emergence or worsening of cognitive and behavioral difficulties represents one of the most frequent and challenging outcomes following ABI in childhood. Estimates suggest that between one-third and three-quarters of affected children exhibit clinically significant behavioral symptoms within the first year after the injury [11]. Secondary attention-deficit/hyperactivity disorder, together with problems in emotional regulation and self-control, are particularly prevalent and concerning, as they may increase vulnerability to subsequent injuries and contribute to social and legal complications [5,12,13]. In the adulthood, a childhood ABI may lead to substance misuse, psychiatric problems, criminal behavior and unemployment [3,5,6,13]. Neurocognitive, behavioral and adaptive challenges affecting children with ABI may negatively impact parents and the family system, due to change in lifestyle and the need to develop additional skills to support their children, which may cause significant physical and emotional burden [14,15]. Families have reported difficulties accessing appropriate treatments to address their child’s challenging behaviors [16]. In particular, disadvantaged families may disproportionately experience the burden associated with ABI, as they may experience greater struggles to access needed services for their child or themselves, which may exacerbate injury-related stress [17,18]. Thus, there is an imperative need for accessible behavioral interventions [19]. Nevertheless, within the field of clinical neuropsychology, evidence-based approaches and services for addressing ongoing and emerging difficulties in children with brain injury remain scarce [20,21].

Many deficits in neurocognitive, behavioral and social functioning of pediatric ABI have been linked to impairments in executive functions (EF) [1,3,4,22–27]. EF are divided into two categories, hot and cold. Hot EF refer to emotion regulation, social skills and affective decision-making, while cold EF include working memory, cognitive flexibility, strategic and organizational abilities, goal setting, problem solving and behavior monitoring [28,29]. EF mediate the effect of the brain injury on social adaptive and academic functioning [1,3,6,22–27,30,31]. Treatments for pediatric ABI populations commonly aim to improve hot and cold EF. [32] Nevertheless, previous research [33–38] has indicated that many children are not provided with the recommended interventions to sustain recovery. This could be due to either barriers in intervention accessibility (geographical reasons, time and economic demands on health care facilities and families) or limited awareness and willingness on the part of patients and families to address these issues [34,39]. Additionally, as many as 75.00% of children fail to receive the needed support for behavioral difficulties [40,41]. To support rehabilitation delivery in this population, a variety of remote, telehealth interventions have been developed over the past two decades, allowing children to receive higher therapy dose [39,42–49].

One of the best validated interventions is the ‘Teen Online Problem Solving’ (TOPS), a technology-assisted program developed in the USA in 2006 by Wade and colleagues [19]. The intervention was initially created to address hot and cold EF problems in adolescents with TBI only but, subsequently, it was adapted for the broader population of adolescents with ABI. The program aims to enhance problem-solving within the family context, drawing on the problem-solving framework of D’Zurilla and colleagues [50,51] and the collaborative family problem-solving model proposed by Robin and Foster [52]. Specifically, the TOPS program addresses organizational, self-regulation and communication challenges in everyday settings, adopting a contextual and personalized rehabilitation approach, which has been found to be more effective in terms of generalization of the effects than the drill-based training modality [45]. Metacognitive strategies to support self-monitoring are also taught, which seems to lead to more cognitive gains [53,54].

Previous research [55,56] found the TOPS program tested in the USA to be feasible and useful and highlighted its significant effects on the adolescents’ functioning (EF, psychological well-being and behavior), parents’ functioning (psychological well-being) and even parent-child conflict [19,45,55,57–60]. The program has been recognized as an evidence-based treatment and a standard of care for executive dysfunction and behavioral problems following ABI [44].

In Italy, there is a lack of remote interventions addressing cognitive or behavioral issues in children with neurological conditions. With respect to children with ABI, prior to 2020 only one remote intervention [61,62] addressing cognitive functions was delivered, but without a focus on everyday EF and behavioral aspects; no other intervention has been reported prior to 2023 [63,64].

In 2018–2019 our research group translated and adapted to the Italian context the original TOPS program, developing the Italian Teen Online Problem Solving (I-TOPS) intervention [39]. While maintaining the same structure as the original TOPS, I-TOPS differs in certain specific content to reflect cultural differences between the Italian and American contexts [39]. To examine the program in the Italian context, in 2021–2023 we conducted a double-blind, phase-II randomized controlled trial (RCT) to test I-TOPS feasibility and efficacy. To reach this goal, the I-TOPS program was compared with an active control training program (named ‘wellness intervention’) having the same duration but omitting problem-solving related content. The research protocol has been registered with Clinicaltrials.gov with identifier NCT05169788 and has been published in a scientific journal [65].

The aim of this study is to present and discuss the feasibility and training satisfaction of the I-TOPS program compared to the wellness intervention in adolescents with ABI aged 11–19 years. To be consistent with extant literature, feasibility outcomes taken from previous studies [66,67] on remote cognitive interventions for this specific population were considered. The feasibility of the training was evaluated through 4 criteria (accessibility, training adherence, technical smoothness and training satisfaction); the feasibility of study and procedures was assessed through 5 criteria (participation willingness, participation rates, loss to follow-up, assessment timescale and assessment procedures). Indeed, before assessing the efficacy of the I-TOPS intervention, it is crucial to first identify potential obstacles to trialing a new intervention practice and explore possible solutions, assess adherence to both the study protocol and the intervention itself, draw preliminary conclusions about the potential for generalization and determine whether any feasibility issues could impact future analyses of efficacy.

This study could provide important information on the possibility of introducing remote interventions in countries with limited investment and implementation in the telerehabilitation field and could inform the Italian healthcare system on viable practices to include in the care path of adolescents with ABI. Due to the length of the I-TOPS program (approximatively 6 months) compared to other remote rehabilitation interventions for EF previously used with young patients with ABI [32,61,62,68,69], findings of this study could gather knowledge on the acceptability of delivering prolonged interventions outside the USA. Ensuring that the program is feasible and acceptable to patients and families provides a crucial foundation for clinical implementation within the Italian healthcare system. Furthermore, this study involved adolescents in the chronic phase following an ABI (≥ 1year post-injury), who were dealing with everyday challenges in their natural environment and typically lacked access to specialized interventions offered by the healthcare system, often resulting in a gap in care. By investigating a contextualized intervention approach, focused on personalized everyday challenges, this study may provide valuable insights into innovative and targeted rehabilitation strategies for a recovery phase that currently lacks specialized and evidence-based practices.

## Methods

### Study design

We conducted a RCT including 2 parallel intervention arms, i.e., I-TOPS intervention and wellness intervention, and 3 assessment time-points, i.e., baseline pre-intervention assessment (T0), immediate post-intervention assessment (T1) and 6-month follow-up after intervention end (T2). Participating adolescents were randomized into the 2 study groups in a 1:1 ratio. The two interventions presented with the same structure, duration and web-based platform but had different contents: in particular, the wellness intervention omitted contents related to problem-solving in order to constitute an active control treatment. The trial was registered on 23 December 2021 in ClinicalTrials.gov, with identifier NCT05169788. The study protocol [65] is available at: https://www.researchprotocols.org/2025/1/e64178/citations [10.2196/64178].

### Eligibility criteria

Inclusion criteria were:

- age between 11:00 and 19:11 years at recruitment;
- diagnosis of non-progressive ABI (TBI, stroke, brain inflammation/infection, anoxia/hypoxia etc.) in the chronic phase (at least one year after the event);
- Full Scale Intellectual Quotient ≥ 70 assessed within one year prior to recruitment;
- adolescent and family’s proper comprehension and speaking abilities in Italian;
- having a personal computer and access to the Internet in the everyday context;
- familiarity of the adolescent and/or the family with basic computer and Internet literacy;
- at least one parent or guardian living with the adolescent demonstrating willingness to participate in the intervention.

Exclusion criteria were:

- a diagnosis of global developmental delay or sensory impairments prior to the ABI;
- a diagnosis of photosensitive epilepsy;
- a history of family abuse;
- a history of psychiatric hospitalization;
- concomitant psychological or neuropsychological intervention.

### Participant recruitment and study procedure

Eligible subjects were identified by the staff members responsible for the study (A.B. and C.C.) in conjunction with the referring physicians from medical records on pediatric ABIs at Scientific Institute IRCCS E. Medea, Bosisio Parini, Lecco, Italy. The research staff contacted families or the adolescents themselves (if of age), either at the Center or by phone, to explain the intervention and study procedures, including assessment timelines, intervention duration, overall objectives, required equipment and other relevant details. No information on group allocation or differences between the I-TOPS and wellness interventions was given, with the aim of reducing user expectation and biased results. For families who expressed willingness to participate in the study, the research staff assessed inclusion criteria and identified the participating parent when both parents were not available. Written informed consent were required for study participation: both parents were asked to read and sign the ‘Participant information sheet and informed consent for research participation’ to consent participation in the study and the ‘Privacy policy statement and consent’ to accept the Institute’s data management policy (see paragraph ‘Ethical considerations and data protection’). After enrollment, adolescents and parents completed the baseline evaluation and were subsequently randomized into the 2 study groups. Participants were not allowed to switch groups; however, each adolescent was permitted to pause participation at any time for family, personal, medical, academic or other reasons. Additionally, to promote adherence, some flexibility in scheduling intervention activities was allowed to accommodate family commitments and holidays or breaks. For more detail, see the ‘Training adherence’ criterion among feasibility criteria. No changes to methods after trial commencement were made. All data were collected at Scientific Institute IRCCS E. Medea, Bosisio Parini, Lecco, Italy.

The study started on 26^th^ February 2021 and ended on 28^th^ February 2023. Recruitment started on 27^th^ December 2021 and ended on 21^st^ February 2022, when the expected study sample size was reached. The last follow-up evaluation was performed on 24^th^ February 2023.

This study was conducted in accordance with CONSORT guidelines for non-pharmacological interventions [70,71].

### Randomization and blinding

Adolescents were randomized into the 2 study arms after completion of the informed consent and assessment of baseline measures. The randomization procedure was carried out by a researcher of the Institute, who was independent from the research staff responsible for recruiting participants. To perform randomization, the Microsoft Excel randomization tool was used. A sealed envelope containing the participant’s group assignment was provided to designated study staff members (A.B. and C.C.) to ensure allocation concealment and prevent selection bias or subversion of the randomization sequence. The sealed envelope was then handed over by the research staff to the psychologists conducting the interventions, who held regular videocalls with participants via Google Meet. Psychologists were not blinded to group allocation in order to allow for clinical supervision, patient interaction and treatment fidelity monitoring. Other research staff, assessors performing outcome evaluations, adolescents and their families were blinded to group assignment.

Specifically, after the baseline assessment (T0) of each patient, the psychologists contacted the patient’s parents (if the patient was a minor) or the patient themselves (if of legal age) to provide instructions regarding the subsequent phases of the study, based on the assigned group. Therefore, the psychologists and the independent researcher who generated the allocation sequence were the only individuals aware of the treatment group assignments. The psychologists were appropriately instructed not to share this information with other team members and to maintain a strict separation of roles. To limit performance bias, the psychologists received weekly supervision from a qualified psychotherapist who held a master’s degree in clinical psychology, a specialization in cognitive-behavioral psychotherapy and certification in clinical neuropsychology. This was done to ensure treatment fidelity, maintain quality standards in treatment delivery and contain potential expectation effects. To limit detection bias, the evaluators responsible for assessments at time points T0, T1 and T2 remained blinded to the participants’ group assignments and were limited to administering only the evaluations specified in the protocol. They received specific training not to ask participants about study-related activities and were instructed to communicate this requirement at the start of each assessment session.

Although the psychologists were the only personnel directly involved in the project who were unblinded to patient allocation, they were also members of the Institute’s clinical staff and could potentially have shared information with the patients’ referring physicians. This situation might have indirectly allowed some uncontrolled information to reach the research team. Therefore, the study was classified as a double-blinded rather than a triple-blinded RCT. Emergency unblinding was never required.

### Sample size and power calculation

The sample size of the study was determined through power analysis performed by using G*Power3 Software [72,73]. An a priori power analysis was conducted to estimate the required sample size, based on the specified alpha level, desired statistical power and expected effect size. We estimated a small effect size = 0.20 for our study on I-TOPS efficacy. This estimate was informed by a previous meta-analysis [45] on the original American TOPS program, which reported an average small-to-moderate effect size (Hedges’ g = 0.37). To be conservative, given the high variability in effect sizes across the studies included in that meta-analysis, we used a small effect size estimate for the power calculation in the present study. With an estimated correlation of 0.50 between repeated measures and an alpha level set at *p* < 0.05, a sample size of 21 subjects per group was needed to obtain 80% of power with a 2 groups x 3 time-points (T0, T1 and T2) design.

### Interventions

This study compared 2 interventions: the I-TOPS (G1) and the wellness intervention (G2). For both interventions, before starting the training, a psychologist of the research staff contacted each participating adolescent and family by telephone to provide instructions on how to access the intervention website and connect to Google Meet calls. Pre-training meetings had a duration of approximatively 45 minutes.

Psychologists delivering interventions underwent specialized training on intervention content and administration and were required to adhere to a detailed procedure provided in a treatment manual developed by Dr. Shari Wade and her research team, who developed the original TOPS intervention, to ensure treatment fidelity.

A detailed description of the I-TOPS and wellness intervention is provided in the paragraphs below. Of note, the I-TOPS and wellness intervention online platforms shared the same graphics, were developed by the same engineering team and relied on identical access procedures and technical features. This ensured that no differences between the two programs could be attributed to platform-related aspects.

For an in-depth look at the content of the I-TOPS and wellness intervention sessions, see the clinical trial protocol [65].

#### I-TOPS intervention (experimental intervention group, G1).

In the TOPS intervention, the adolescent and her/his parents were required to complete online learning sessions in the ecological setting. Specifically, they had to read sessions’ didactic contents on the ABI and related consequences, learn problem-solving skills (organization/planning, social interactions and emotion control), watch videoclips of teens modeling these problem-solving skills and perform ad-hoc exercises giving them additional practice with the concepts. Then, they were required to meet synchronously with a psychologist with expertise in cognitive-behavioral therapy biweekly to discuss and review website content and apply the steps of problem-solving to the adolescent’s or family’s everyday challenges. Ten core online learning sessions were assigned to all participating family members, whereas other supplemental sessions were only assigned to adolescents and families with concerns in the following areas: sibling concerns, parent concerns related to other children, marital communication, talking with a teen, crisis management, sleep, planning for after high school, family coping and epilepsy. A new session was required to be completed approximately every 15 days, with online session duration ranging from 30 to 45 minutes. After completing the online module, the adolescent and parent met with the psychologist via the Google Meet platform to review the online content and develop problem-solving plans. A maximum of 2 supplemental sessions could be scheduled for each adolescent and family. Therefore, the program took an average of 6 months to complete (range: 5–7 months).

While the I-TOPS program followed a structured protocol with predefined contents, the problem-solving component was tailored to address the personalized, real-life issues of the adolescents and their families. For this reason, prior to beginning the intervention, the psychologist held an online meeting with the adolescents and their families to identify specific challenges of daily life in the domains of organization and planning, social interactions and emotion regulation that they intended to work on during the intervention. Additionally, adolescents and families could identify additional goals during the intervention.

#### Wellness intervention (active control training, G2).

The wellness intervention focused on health and wellness content only, with no discussion or support of problem-solving abilities. The wellness program had the same structure and duration as the I-TOPS program, thus including 10 core sessions and up to 2 supplemental sessions. Each session had to be completed approximately every 15 days and lasted about 20–30 minutes (they did not have contents related to strategies to apply in everyday activities, thus had a shorter duration than the one of I-TOPS). After completing each online module, a 15-minute online meeting with the psychologist was scheduled via the Google Meet platform to promote knowledge of healthy lifestyle behaviors aimed at improving wellness, as well as to support training compliance. Similar to the I-TOPS intervention, completion of the 10 core and any supplemental sessions took each adolescent and family approximatively 6 months.

The use of an active control group allowed us to control for placebo effects and isolate the specific effects of problem-solving training delivered through the I-TOPS intervention. In fact, the wellness group, by ensuring a level of engagement comparable to that of the I-TOPS group, unlike a passive control, enabled us to account for potential placebo-related effects.

### Feasibility criteria

To assess the feasibility of the training and study design and procedures, we adopted 9 outcome measures used in previous studies [66,67] on remote cognitive interventions for adolescents with ABI. This was with the aim of using defined a-priori criteria and allowing comparisons. The 9 criteria used are described below.

To assess the feasibility of the training (I-TOPS or wellness intervention) the following 4 outcome measures were used:

- Accessibility: number and percentage of adolescents who required further instructions to understand training content and session-related tasks when at home. Criterion for success: the criterion was considered met if 90.00% of families understood training content and session-related tasks when at home, after maximum 2 explanations of sessions’ content and activities by the psychologist. If 3 or more explanations were requested, the criterion was considered not satisfied.
- Training adherence: number and percentage of the 10 core sessions completed during the expected training period. Data on drop-outs were included in the calculation, which was carried out considering the average training completion in each group. Criterion for success: criterion fulfillment was defined as the completion of an average of 80.00% of the 10 core treatment sessions within 5 months in each treatment group. For the remaining 20.00% of sessions, an additional month (i.e., 2 extra weeks per session) was allowed beyond the scheduled timeframe to provide flexibility for family commitments, vacation periods or temporary interruptions.
- Technical smoothness: number and percentage of adolescents who experienced technical issues with the training material and platform persisting for 2 or more weeks and which caused the training to be interrupted and the timeline to be extended. Criterion for success: 100.00% of adolescents did not experience significant technical issues. Drop-outs were included in the evaluation of criterion fulfillment.
- Training satisfaction: an ad-hoc 19-item questionnaire, developed for the original TOPS program and already used in previous studies [55,56] on program feasibility, was administered to assess training satisfaction. Questions addressed included perceived changes in one’s problem-solving skills and psychological wellness, satisfaction with the program in terms of helpfulness, enjoyment and relevance of contents and website ease of use. Responses were provided on a Likert scale ranging from 1 (strongly disagree) to 4 (strongly agree). Good satisfaction was conveyed by a total score of 57 (neutral score) or greater (positive score). The questionnaire was administered to both groups, with no differences in item wording. Criterion for success: 80.00% of adolescents and families provided a neutral or positive total score.

Feasibility of study design and procedures was assessed using the following 5 outcome measures:

- Willingness to participate: number of adolescents and families who agreed to participate among those who were contacted for study enrollment. Criterion for success: 75.00% of eligible adolescents and families agreed to take part in the study; the criterion was based on the pre-randomization data.
- Participation rates: number and percentage of adolescents who completed one or more sessions of the assigned intervention, without dropping out between the baseline assessment (T0) and the start of the training. Criterion for success: 80.00% of participants who agreed to partake actually participated in the study.
- Loss to follow-up: number of adolescents who did not complete post-training (T1) and 6-month follow-up assessment (T2). Criterion for success: less than 20.00% of participants did not complete post-training assessment (T1) and 6-month follow-up assessment (T2). Participants who dropped out of the training were excluded from the evaluation of criterion fulfillment.
- Assessment procedures: number and percentage of adolescents and families for whom outcome measures were collected on the 3 assessment time points (T0, T1 and T2). A secondary analysis was conducted on the collection of adolescent-related and parent/family-related outcome measures separately, to assess any potential differences in reporting. Adolescent-related outcome measures were: Child Behavior Checklist 6–18, [74] Youth Self-Report 11–18, [74] Behavior Rating of Executive Function 2-self report form, [75] Behavior Rating of Executive Function 2-parent form, [75] Jansari Assessment of Executive Functions for Children [76] and A Developmental Neuropsychological Assessment, Second Edition [77]). Parent/family-related outcome measures were: Beck Anxiety Inventory, [78] Parenting Stress Index [79] and Symptom Checklist-90 [80]. Criterion for success: at each assessment time point, 90.00% of all the outcome measures for each participant had to be collected. Participants who did not complete the T1 and/or T2 assessments were excluded from the evaluation of criterion fulfillment at the respective time point(s).
- Assessment timescale: the number of adolescents whose follow-up data were collected within one week of training completion for the post-training evaluation (T1) and within two weeks after the 6-month post-training period for the follow-up evaluation (T2) was considered in the evaluation of this outcome. To monitor adherence to the T1 and T2 timelines for questionnaire return, the return-postmark date was used as the operational reference for determining whether assessments were completed within the required timeframe, acknowledging that postal processing may introduce an approximate one-day delay. Adherence to performance-based assessments conducted via Google Meet was considered met when assessments were performed within the scheduled time interval. Criterion for success: for 100.00% of the adolescents the expected timelines for assessments were met. Participants who did not complete the T1 and/or T2 assessments were excluded from the evaluation of criterion fulfillment at the respective time point(s).

### Outcome measure to test preliminary efficacy

A well-known and validated outcome measure included in this RCT, namely the total score from the Child Behavior Checklist 6–18 [74], which represents the study primary outcome for the evaluation of efficacy, was used to assess pre- and post-intervention differences within each study group. This measure evaluates the psychological adjustment and behavioral functioning of children and adolescents.

### Ethical considerations and data protection

This study was approved by the Ethic Committee of Scientific Institute, IRCCS E. Medea, Bosisio Parini, Lecco, Italy, on 21 January 2021 with identifier ‘08/21-CE’ (conditioned favorable opinion) and 26 February 2021 with identifier ‘19/21–CE’ (final favorable opinion) and was conducted in accordance with the protocol and the principles of the 1964 Declaration of Helsinki. Each participating adolescent (if of age) or their parents or guardians (if under the age of consent) were required to sign a ‘Participant information sheet and informed consent for research participation’ to consent to participate in the study along with a ‘Privacy policy statement and consent’ to adhere to the Institute’s data management policy.

To keep participants pseudonymized, a unique trial number was assigned to each of them. Names and addresses of participants associated with their trial numbers were stored securely and all data were inserted in a password-protected database.

### Statistical analyses

Descriptive statistics (means, standard deviations, frequencies and percentages) were used to characterize the demographic and clinical features of the sample and to report satisfaction with the feasibility outcomes. Inferential statistics were used to compare continuous variables, using the independent-samples t test or Mann–Whitney U test for bewteen-group comparisons and the paired-samples t-test or Wilcoxon signed-rank test for within-group comparisons, as appropriate. Categorical variables were compared using the chi square test or Fisher’s exact test. Correlations were assessed using Pearson’s correlation coefficient.

Attrition was taken into account in the present feasibility study, as drop-out rates represented key data for assessing feasibility outcomes; therefore, drop-outs did not result in an underpowered feasibility study. Preliminary efficacy was evaluated separately within each group using the paired-sample Wilcoxon signed-rank tests on the Child Behavior Checklist 6–18 total score [74] (which is the study’s primary outcome). Time (T0 vs. T1) was treated as the within-subject factor. The Wilcoxon signed-rank test was chosen because the assumption of normality was violated for the Child Behavior Checklist 6–18 total score [74] at T0 (Kolmogorov Smirnov (42) = 0.14, *p* = 0.03).

Level of significance was set at *p* < 0.05. SPSS V.29.0.1.0 [81] was used to perform analyses.

## Results

### Recruitment

Among the 81 patients eligible to partake in the study, 50 (61.73%) agreed to participate. 8 adolescents were excluded by study staff as they did not fully meet eligibility criteria. Therefore, a sample of 42 adolescents was reached, in line with sample size established by the power analysis.

**Fig 1:**
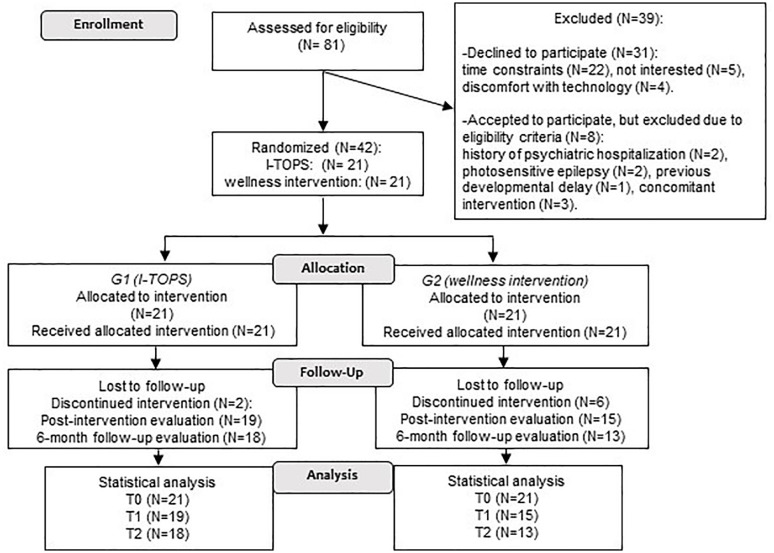
CONSORT flow diagram.

CONSORT flow diagram is depicted in Fig 1.

Twenty-four of the adolescents were males (57.14%) and the most common diagnosis was TBI (50.00%), followed by arteriovenous malformation (19.05%) and central nervous system infection/inflammation (16.67%). The distribution of etiologies in the present sample, with half of the participants having sustained a TBI, is consistent with previous epidemiological data on pediatric brain injuries in Italy [82]. The average FSIQ, assessed after the injury and within one year prior to study initiation, was 93.26 (SD = 14.25) across the entire sample, falling within the average range. No differences in FSIQ were found between the I-TOPS and the wellness intervention (I-TOPS: 95.00, SD = 14.13; wellness: 90.38, SD = 16.51, t(40) = 0.79, *p* = 0.44). The main parent who participated in the intervention was the mother (85.71%) and the most common level of parental education was high school or less (69.05%). 64.29 of parents were employed full time and 90.48% were married or lived with someone.

Among the 42 adolescents enrolled in the study, 21 were assigned to and received the I-TOPS intervention, and 21 were assigned to and received the wellness intervention. All 42 adolescents began the training and none were lost after randomization. Data from all 42 participants, regardless of adherence to the training, were included in the analysis of feasibility outcomes. Complete data on participants and study results are provided in the Supporting Information, in accordance with the PLOS data policy.

Table 1 presents the demographic, intellectual and clinical characteristics of the adolescents, as well as the demographic characteristics of their families, for both the entire sample and stratified by intervention group.

**Table 1:** Demographic, intellectual and clinical characteristics of the adolescents and demographic characteristics of the families in the whole sample and by intervention group.

	WHOLE GROUP (n = 42)*		I-TOPS GROUP (G1) (n = 21)*		WELLNESS GROUP (G2) (n = 21)*		
	n (%)	M (SD)	n (%)	M (SD)	n (%)	M (SD)	*p*
**Adolescents’ demographic variables**							
Gender (male)	24 (57.14%)		13 (61.90%)		11 (52.39%)		0.53
Age (years)		14.60 (2.76)		14.71 (2.77)		14.48 (2.80)	0.78
**Adolescents’ intellectual functioning**							
FSIQ score		92.26 (14.25)		95.00 (14.13)		91.52 (14.51)	0.44
FSIQ ranges							0.92
Borderline (70–79)	9 (21.43%)		4 (19.05%)		5 (23.81%)		
Low average (80–89)	8 (19.05%)		4 (19.05%)		3 (14.03%)		
Average (90–109)	20 (47.62%)		10 (47.62%)		11 (52.38%)		
High average or superior (≥110)	5 (11.90%)		3 (14.29%)		2 (9.52%)		
**Adolescents’ clinical variables**							
Diagnosis							0.95
-Head trauma	21 (50.00%)		11 (52.39%)		10 (47.62%)		
-Arteriovenous malformation rupture	8 (19.05%)		3 (14.29%)		5 (23.81%)		
-Brain infection	7 (16.67%)		4 (19.05%)		3 (14.29%)		
-Stroke	4 (9.52%)		2 (9.52%)		2 (9.52%)		
-Cerebral anoxia	2 (4.76%)		1 (4.76%)		1 (4.76%)		
Age at diagnosis (years)		8.76 (4.24)		9.57 (4.47)		7.95 (3.93)	0.22
**Drop out(s)**							0.12
n (%)	8 (19.05%)		2 (9.52%)		6 (28.57%)		
**Parent-related variables**							
Parent who participated in intervention							0.72
-Mother only	36 (85.71%)		18 (85.71%)		18 (85.71%)		
-Father only	3 (7.14%)		1 (4.76%)		2 (9.52%)		
-Mother and Father	3 (7.14%)		2 (9.52%)		1 (4.76%)		
Parent education							0.74
-High school diploma or less	29 (69.05%)		14 (66.67%)		15 (71.43%)		
-College or more	13 (30.95%)		7 (33.33%)		6 (28.57%)		
Parent employment status							0.26
-Full-time	27 (64.29%)		11 (52.39%)		16 (76.19%)		
-Part-time	10 (23.81%)		7 (33.33%)		3 (14.29%)		
-Not currently employed	5 (11.90%)		3 (14.29%)		2 (9.52%)		
Parent marital status							0.29
-Married or living with someone	38 (90.48%)		20 (95.24%)		18 (85.71%)		
-Not living with someone	4 (9.52%)		1 (4.76%)		3 (14.29%)		
SES		61.19 (16.99)		60.95 (15.46)		61.43 (18.78)	0.93

*drop-outs included. FSIQ = full scale intellectual quotient; SES = socio-economic status.

Within the whole sample and in the I-TOPS group, completers and non-completers did not differ in any clinical, intellectual and demographic variables related to the adolescent or the family (all *p* ≥ 0.11). However, non-completers in the wellness group were significantly older (M = 16.67; SD = 2.42) than completers (M = 13.60; SD = 2.50) (*p* < 0.02). No significant differences were found in the wellness group for other clinical, intellectual and demographic variables related to the adolescent or the family (all *p* ≥ 0.31). Demographic, intellectual and clinical characteristics of the adolescents and demographic characteristics of the families by completion status are summarized in Table 2 for the whole group and in Table 3 for I-TOPS and wellness intervention groups separately.

**Table 2:** Demographic, intellectual and clinical characteristics of the adolescents and demographic characteristics of the families by completion status in the whole group.

	Whole group (n = 42)				
	Completers (n = 34)		Non completers (n = 8)		
	n (%)	M (SD)	n (%)	M (SD)	*p*
**Adolescents’ demographic variables**					
Gender (male)	19 (55.89%)		5 (62.50%)		0.53
Age (years)		14.24 (2.75)		15.75 (2.71)	0.11
**Adolescents’ intellectual functioning**					
FSIQ score		93.44 (14.07)		92.50 (15.97)	0.87
FSIQ ranges					0.98
Borderline (70–79)	7 (20.59%)		2 (25.00%)		
Low average (80–89)	6 (17.65%)		1 (12.50%)		
Average (90–109)	17 (50.00%)		4 (50.00%)		
High average or superior (≥110)	4 (11.76%)		1 (12.50%)		
**Adolescents’ clinical variables**					
Diagnosis					0.37
-Head trauma	16 (47.06%)		5 (62.50%)		
-Arteriovenous malformation rupture	6 (17.65%)		2 (25.00%)		
-Brain infection	7 (20.59%)		0 (0.00%)		
-Stroke	4 (11.76%)		0 (0.00%)		
-Cerebral anoxia	1 (2.94%)		1 (12.50%)		
Age at diagnosis (years)		8.50 (4.36)		9.75 (4.56)	0.39
**Parent-related variables**					
Parent who participated in intervention					0.44
-Mother	28 (82.35%)		8 (100%)		
-Father	3 (8.82%)		0 (0.00%)		
-Mother and Father	3 (8.82%)		0 (0.00%)		
Parent education					0.69
-High school diploma or less	23 (67.65%)		6 (75.00%)		
-College or more	11 (32.35%)		2 (25.00%)		
Parent employment status					0.70
-Full-time	21 (61.76%)		6 (75.00%)		
-Part-time	9 (26.47%)		1 (12.50%)		
-Not currently employed	4 (11.76%)		1 (12.50%)		
Parent marital status					0.75
-Married or living with someone	31 (91.18%)		7 (87.50%)		
-Not living with someone	3 (8.82%)		1 (12.50%)		
SES		60.29 (17.14)		65.00 (16.90)	0.48

Between group-differences were calculated through χ^2^ or T-Test. FSIQ = full scale intellectual quotient; SES = socio-economic status.

**Table 3:** Demographic, intellectual and clinical characteristics of the adolescents and demographic characteristics of the families by completion status in the I-TOPS and wellness intervention groups.

	I-TOPS GROUP (G1) (n = 21)					WELLNESS GROUP (G2) (n = 21)				
	Completers (n = 19)		Non completers (n = 2)			Completers (n = 15)		Non completers (n = 6)		
	n (%)	M (SD)	n (%)	M (SD)	*p*	n (%)	M (SD)	n (%)	M (SD)	*p*
**Adolescents’ demographic variables**										
Gender (male)	11 (57.89%)		2 (100.00%)		0.24	8 (53.33%)		3 (50.00%)		0.89
Age (years)		14.84 (2.89)		13.50 (0.71)	0.53		13.60 (2.50)		16.67 (2.42)	**0.02**
**Adolescents’ intellectual functioning**										
FSIQ score		95.05 (14.08)		94.50 (20.51)	0.95		91.40 (14.28)		91.83 (16.46)	0.91
FSIQ ranges					0.60					0.58
Borderline (70–79)	4 (21.05%)		0 (0.00%)			3 (20.00%)		2 (33.33%)		
Low average (80–89)	3 (15.79%)		1 (50.00%)			3 (20.00%)		0 (0.00%)		
Average (90–109)	9 (47.37%)		1 (50.00%)			8 (53.33%)		3 (50.00%)		
High average or superior (≥110)	3 (15.79%)		0 (0.00%)			1 (6.67%)		1 (16.67%)		
**Adolescents’ clinical variables**										
Diagnosis					0.73					0.31
-Head trauma	9 (47.37%)		2 (100.00%)			7 (46.67%)		3 (50.00%)		
-Arteriovenous malformation rupture	3 (15.79%)		0 (0.00%)			3 (20.00%)		2 (33.33%)		
-Brain infection	4 (21.05%)		0 (0.00%)			3 (20.00%)		0 (0.00%)		
-Stroke	2 (10.53%)		0 (0.00%)			2 (13.33%)		0 (0.00%)		
-Cerebral anoxia	1 (5.26%)		0 (0.00%)			0 (0.00%)		1 (16.67%)		
Age at diagnosis (years)		9.26 (4.59)		12.50 (0.71)	0.37		7.60 (3.56)		8.83 (5.00)	0.53
**Parent-related variables**										
Parent who participated in intervention					0.83					0.50
-Mother	16 (84.21%)		2 (100.00%)			12 (80.00%)		6 (100.00%)		
-Father	1 (5.26%)		0 (0.00%)			2 (13.33%)		0 (0.00%)		
-Mother and Father	2 (10.53%)		0 (0.00%)			1 (6.67%)		0 (0.00%)		
Parent education					0.60					0.45
-High school diploma or less	13 (68.42%)		1 (50.00%)			10 (66.67%)		5 (83.33%)		
-College or more	6 (31.58%)		1 (50.00%)			5 (33.33%)		1 (16.67%)		
Parent employment status					0.26					0.64
-Full-time	10 (52.63%)		1 (50.00%)			11 (73.33%)		5 (83.33%)		
-Part-time	7 (36.84%)		0 (0.00%)			2 (13.33%)		1 (16.67%)		
-Not currently employed	2 (10.53%)		1 (50.00%)			2 (13.33%)		0 (0.00%)		
Parent marital status					0.74					0.84
-Married or living with someone	18 (94.74%)		2 (100.00%)			13 (86.67%)		5 (83.33%)		
-Not living with someone	1 (5.26%)		0 (0.00%)			2 (13.33%)		1 (16.67%)		
SES		60.53 (15.45)		65.00 (21.21)	0.71		60.00 (19.64)		65.00 (17.61)	0.60

Between group-differences were calculated through χ^2^/Fisher’s test or T-Test/Mann-Whitney U. FSIQ = full scale intellectual quotient; SES = socio-economic status.

### Feasibility outcome measures

Data on feasibility outcomes are presented below and summarized in Table 4.

**Table 4:** Results on feasibility outcome measures by intervention group.

	*Feasibility measures*	*Feasibility questions*	*Data collected*	*Feasibility criterion for success*	*Outcome*	*Success*
Training feasibility	accessibility	Do participants understand sessions’ contents and tasks when at home, after the explanation of sessions’ access, contents and activities by the psychologist?	Number of participants who asked for further instruction to understand sessions’ contents and tasks when at home. If the participant required a third explanation via remote call, the criterion was considered unmet.	90.00% of participants understood all the procedure and sessions’ contents. The criterion was considered not to be met if after 2 explanations, participants asked for further ones.	I-TOPS group: 90.47% of participants understood all the procedure and sessions’ contents (19/21). 2 participants required further explanations.	I-TOPS group: YES
					Wellness group: 100.00% of participants understood all the procedure and sessions’ contents (21/21).	Wellness group: YES
	training adherence	Do participants complete all the 10 training sessions during the scheduled training period?	Mean percentage of sessions completed during the scheduled training period.	80.00% of training intervention was completed in the scheduled timeframe. The remaining 20.00% (2 core sessions) could be completed in an extra time of 1 month.	I-TOPS group: average training completion of 91.90%. 2/21 adolescents dropped out during the training, performing 10.00% and 20.00% of training, respectively. Only 1 participant required an extra time of 2 weeks to complete 1 session, but concluded the training in the expected timeframe.	I-TOPS group: YES
					Wellness group: average training completion of 75.24%; 6/21 adolescents dropped out during the training, 4 performing 10.00% and 2 20.00% of the training. 2 participants required an extra time of 2 weeks to complete one session, but concluded the training in the expected timeframe.	Wellness group: NO
	technical smoothness	Are there any technical issues with accessing and using the training program?	Number of participants who encountered technical issues that persisted for 2 weeks or more, thereby generating a training interruption and possibly influencing total training duration.	90.00% of participants were able to perform the training without technical issues.	I-TOPS group: 100.00% of participants were able to perform the training without technical issues. One participant encountered a technical issue as a result of losing their own login credentials. This issue was resolved by contacting the help center and resetting the login credentials, not influencing training duration.	I-TOPS group: YES
					Wellness group: 100.00% of participants were able to complete the training without major technical issues. One participant experienced a problem accessing the videos on the web-based platform, but the issue was quickly resolved by contacting the help center, allowing the training to continue without further interruption.	Wellness group: YES
	training satisfaction	Do participants appreciate the training intervention?	Scores at an ad-hoc satisfaction questionnaire filled in at the end of the training.	80.00% of participants had a neutral total score (= 57) or positive total score (> 57) on the satisfaction questionnaire.	I-TOPS group: 89.47% of participants who concluded the training had a neutral (1/19: 5.26%) or positive total score (16/19: 84.21%) on the questionnaire.	I-TOPS group: YES
					Wellness group: 60.00% of participants who concluded the training (9/15) had a positive total score on the questionnaire.	Wellness group: NO
Feasibility of study design and procedures	participation willingness	What is the participation rate?	Number of subjects who provided consent to participate in the study out of those identified as potentially eligible and contacted.	75.00% of potentially eligible and contacted subjects who agreed to take part in the study.	50/81 eligible participants (61.73%) agreed to partake in the study. 8 subjects were excluded by clinicians, because they did not fully meet the inclusion criteria.	Whole group: NO (pre-randomization data)
	participation rates	Do all eligible participants who agreed to partake actually perform the entire training intervention?	Number of enrolled participants who actually performed the training intervention and number of participants who abandoned the training before concluding all the core sessions.	80.00% of participants completed the entire training program.	I-TOPS group: 19/21 (90.47%) enrolled participants completed the entire training program. 9.52% of participants failed to complete the training.	I-TOPS group: YES
					Wellness group: 15/21 (71.43%) enrolled participants completed the entire training program. 28.57% of participants failed to complete the training.	Wellness group: NO
	loss to follow-up	Was the loss to follow-up acceptable?	Number of patients who did not drop out of the training and completed post-training and 6-month follow-up assessments.	Less than 20.00% of participants failed to undergo post-training and 6-month follow-up assessments.	I-TOPS group: 100.00% (19/19) of participants who finished the intervention completed immediate post-training assessment. 94.74% (18/19) underwent follow-up assessment.	I-TOPS group: YES
					Wellness group: 100.00% (15/15) of participants who finished the intervention completed immediate post-training assessment. 86.67% (13/15) underwent follow-up assessment.	Wellness group: YES
						
	assessment procedures	Can all outcome data be collected without any problems?	Percentage of outcome measures collected without any problems (missing data, technical issues related to assessment tools etc.) on the 3 assessment time points (T0, T1 and T2) for each participant. A secondary analysis was conducted on the collection of adolescent-related and parent/family-related outcome measures separately, to assess any potential differences in reporting.	90.00% of all outcome measures of each participant were collected on each assessment time point.	I-TOPS group: T0: for 71.43% (15/21) of participants, at least 90.00% of outcome measures, including both adolescent- and parent/family-related outcomes, were collected. However, for 95.24% (20/21) of participants, at least 90.00% of adolescent-only outcome measures were collected. T1: for 73.68% (14/19) of participants who underwent T1 assessment, at least 90.00% of outcome measures, including both adolescent- and parent/family-related outcomes, were collected. However, for 100.00% (19/19) of participants, at least 90.00% of adolescent-only outcome measures were collected. T2: for 72.22% (13/18) of participants who underwent T2 assessment, at least 90.00% of outcome measures, including both adolescent- and parent/family-related outcomes, were collected. However, for 100.00% (18/18) of participants, at least 90.00% of adolescent-only outcome measures were collected.	I-TOPS group: NO
					Wellness group: T0: for 71.43% (15/21) of participants, at least 90.00% of outcome measures, including both adolescent- and parent/family-related outcomes, were collected. However, for 100.00% (21/21) of participants, at least 90.00% of adolescent-only outcome measures were collected. T1: for 86.67% (13/15) of participants who underwent T1 assessment, at least 90.00% of outcome measures, including both adolescent- and parent/family-related outcomes, were collected. However, for 100.00% (15/15) of participants, at least 90.00% of adolescent-only outcome measures were collected. T2: for 84.62% (11/13) of participants who underwent T2 assessment, at least 90.00% of outcome measures, including both adolescent- and parent/family-related outcomes, were collected. However, for 100.00% (13/13) of participants, at least 90.00% of adolescent-only outcome measures were collected.	Wellness group: NO
	assessment timescale	Can outcome measures be collected within 1 week after completion of training for post-training evaluation (T1) and within 2 weeks after the 6-month period after the end of training for follow-up evaluation (T2)?	Number of participants whose outcome measures were collected within a week after the training and within 2 weeks after the 6-month period after the end of training.	Interval between training end and immediate post-training evaluation (T1) ≤7 days, and between training end and 6-month follow-up evaluation (T2) ≤14 days for 100.00% of participants.	I-TOPS group: at T1 post-training measures of 94.74% (18/19) of participants undergoing T1 assessment have been collected within 1 week after training end. At T2, follow-up measures of 88.89% (16/18) of participants undergoing T2 assessment have been collected within 2 weeks after the 6-month period after the end of training (T2).	I-TOPS group: NO
					Wellness group: at T1 post-training measures of 86.67% (13/15) of participants undergoing T1 assessment have been collected within 1 week after training end. At T2 follow-up measures of 92.31% (12/13) of participants undergoing T2 assessment have been collected within 2 weeks after the 6-month period after the end of training (T2).	Wellness group: NO

### Training feasibility

#### Accessibility.

The criterion was met by both intervention groups. In the I-TOPS group, 90.47% of adolescents (19/21) understood training contents and session-related tasks when remotely performing the intervention. Only 2 adolescents requested further instructions after the 2 initial explanations. In the wellness intervention group, 100.00% of adolescents (21/21) understood training contents and session-related tasks, without requiring any extra explanations.

#### Training adherence.

The criterion was met by the I-TOPS intervention group but not by the wellness intervention group. Specifically, adolescents in the I-TOPS group completed an average of 91.90% of the training within the scheduled time frame. One adolescent required additional 2 weeks to complete a session, due to school commitments. Two adolescents dropped out during the intervention, having completed 10.00% and 20.00% of the program, respectively. In contrast, adolescents in the wellness group completed an average of 75.24% of the training within the scheduled time frame. Six adolescents dropped out during the intervention: 4 completed only 1 out of the total 10 sessions (10.00% completion), and 2 completed 2 out of 10 sessions (20.00% completion).

#### Technical smoothness.

The criterion was met for both groups. All participants in both groups (21/21, 100.00% for I-TOPS and 21/21, 100.00% for wellness group) accessed and performed the training without technical issues persisting for 2 or more weeks.

#### Training satisfaction.

The criterion was met by the I-TOPS group but not by the wellness group (the criterion was calculated only on adolescents and families who completed the interventions). 89.47% of families of the I-TOPS group who completed the program (17/19) assigned either a neutral total score (= 57; 1/19: 5.26%) or a positive total score (≥ 57); 16/19: 84.21%) on the training satisfaction questionnaire, whereas only 60.00% of those of the wellness group (9/15) assigned a positive total score. Differences ≥ 15% between scores in the two groups were found on the following items: 3 (*I know how to handle a crisis when it comes up*), 8 (*I would do the program over*), 11 (*The program met my expectations*), 13 (*The program was useful*), 14 (*I enjoyed the program*), on which adolescents of the I-TOPS group assigned higher scores.

The percentages of positive responses (i.e., ‘agree’ or ‘strongly agree’) on each of the 19 items of the training satisfaction questionnaire, by intervention group, are reported in Table 5.

**Table 5:** Percentages of positive responses on the ‘Satisfaction questionnaire’ by intervention group.

		I-TOPS GROUP (G1) (n = 19)	WELLNESS GROUP (G2) (n = 15)
N. item	Item	Agree or strongly agree (%)	Agree or strongly agree (%)
1	Ho raggiunto gli obiettivi che mi ero posto/a a inizio intervento [*I have reached the goals that I had when I began the program*].	100.00%	100.00%
2	Ho una strategia per gestire i problemi futuri [*I have a plan for handling future problems*].	94.74%	93.33%
3	So come gestire una crisi se/quando mi capiterà [*I know how to handle a crisis when it comes up*].	89.47%	73.33%
4	Mi sento meno stressato/a [*I feel less stressed*].	78.95%	80.00%
5	L’intervento è stato troppo lungo* [*The program was too long*].	78.95%	73.33%
6	L’intervento ha avuto una lunghezza adeguata [*The program had an appropriate length*].	78.95%	73.33%
7	Le informazioni contenute nel programma non sono state utili a me/alla mia famiglia* [*The information included in the program did not apply to me or my family*].	78.95%	73.33%
8	Rifarei l’intervento [*I would do the program over*].	89.47%	60.00%
9	Consiglierei a qualcun altro di partecipare all’intervento [I would recommend the program to others].	100.00%	86.66%
10	Ho fatto dei buoni cambiamenti [*I have made positive changes*].	84.21%	93.33%
11	L’intervento è stato come me lo immaginavo [*The program met my expectations*].	100.00%	80.00%
12	L’intervento è stato d’aiuto [*The program was helpful*].	94.74%	80.00%
13	L’intervento è stato utile [*The program was useful*].	94.74%	73.33%
14	L’intervento mi è piaciuto [*I enjoyed the program*].	94.74%	73.33%
15	15. Il programma è stato facile da usare [*The website was easy to use*].	100.00%	100.00%
16	Il sito web è stato utile [*The website was useful*].	100.00%	100.00%
17	Mi è piaciuto utilizzare il sito web [*I enjoyed using the website*].	100.00%	86.66%
18	Il sito web è stato facile da capire e utilizzare [*The website was easy to understand and navigate*].	100.00%	93.33%
19	I contenuti sono stati interessanti [*The content was relevant*].	94.74%	100.00%

*For negatively worded items, reverse percentage scores were used for the analyses.

### Feasibility of study design and procedures

#### Participation willingness.

This criterion was not met. Participation rate into the study was 61.73%: 50/81 potentially eligible adolescents agreed to partake in the study. Among the 31 adolescents and families who declined participation (38.27%), reasons for declining were: time constraints (n = 22, 70.96%), no interest in participating in a rehabilitation program (n = 5, 16.13%) or discomfort with the technical aspects (n = 4, 12.90%). Of the 50 adolescents who were willing to participate, 8 did not meet all eligibility criteria (history of psychiatric hospitalization: n = 2; photosensitive epilepsy: n = 2; previous diagnosis of developmental delay: n = 1; concomitant psychological or neuropsychological intervention; n = 3) and were thus excluded by the research team.

#### Participation rates.

The criterion was met by the I-TOPS group but not by the wellness group. All participants in both groups who were enrolled in the study actually participated (I-TOPS: 21/21, 100.00%; Wellness:21/21, 100.00%). However, among them, 90.47% of adolescents in the I-TOPS group and 71.43% of those in the wellness group completed the entire training.

#### Loss to follow-up.

The criterion was met both by the I-TOPS and the wellness group. All participants of both groups who concluded the intervention (I-TOPS group: 19/19, 100.00%; wellness group: 15/15, 100.00%) completed immediate post-training assessment (T1). At 6-month follow-up (T2) one drop-out in the I-TOPS group (1/19, 5.26%) and 2 drop-outs in the wellness group (2/15, 13.33%) were registered. Therefore, at T2, 94.74% of patients (18/19) in the I-TOPS group and 86.67% (13/15) in the wellness group underwent the follow-up assessment.

#### Assessment procedure.

Neither group met this criterion when considering all outcome measures, thus including both adolescent-related and parent/family-related outcomes. Specifically, at T0, 71.43% of participants in both groups (15/21 in each group) had at least 90.00% of all adolescent-related and parent/family-related outcome measures completed. However, when examining adolescent-related and parent/family-related outcomes separately, 95.24% of adolescents in the I-TOPS group (20/21) and 100.00% in the wellness group (21/21) had all measures related to adolescent functioning completed. Therefore, the criterion was met by both groups when considering only outcome measures related to the adolescents. At T1 (post-treatment assessment), the criterion was again not met by either group when considering all outcome measures. Specifically, among adolescents who completed the T1 assessment, 73.68% in the I-TOPS group (14/19) and 86.67% in the wellness group (13/15) had all outcome measures completed. However, once again, when considering only measures assessing adolescent functioning, 100.00% of adolescents and parents in both groups completed these measures. At T2, among adolescents who completed the assessment, 72.22% of participants in the I-TOPS group (13/18) and 84.62% in the wellness group (11/13) had all outcome measures completed. When examining adolescent-related outcomes only, the completion rate reached 100.00% in both the I-TOPS group (18/18) and the wellness group (13/13).

#### Assessment timescale.

The criterion was not met in either the I-TOPS or the wellness group. All adolescents in both groups were assessed within one week after signing the informed consent at baseline (T0). However, at T1 (post-training assessment), outcome measures for one adolescent in the I-TOPS group (18/19) and 2 adolescents in the wellness group (13/15) were not collected within one week after the end of training due to the unavailability of the adolescents and their families. As a result, at T1 the assessment timescale was respected in 94.74% of cases in the I-TOPS group and 86.67% in the wellness group. At T2 (follow-up assessment), outcome measures for 2 adolescents in the I-TOPS group (16/18) and one adolescent in the wellness group (12/13) were not collected within 2 weeks following the 6-month post-training period (T2), again due to the unavailability of the adolescents and their families. Therefore, at T2 the assessment timescale was respected in 88.89% of cases in the I-TOPS group and 92.31% in the wellness group.

### Correlations between FSIQ and SES and training adherence and satisfaction

Age at intervention correlated with training adherence in the wellness group (older adolescents showed lower adherence rates) (r = −0.53, *p* = 0.01) but not in the I-TOPS group (r = 0.14, *p* = 0.54). Training adherence corresponds to completion rates, intended as the percentage of the core sessions completed during the scheduled training period by each participant. Age at intervention also correlated with training satisfaction (total score on the satisfaction questionnaire) in the wellness group (r = 0.52, *p* = 0.02), but not in the I-TOPS group (r = −0.14, *p* = 0.53), with older adolescents showing lower satisfaction rates.

Conversely, in both interventions groups, the following demographic and clinical variables did not correlate with training adherence: FSIQ (I-TOPS: r = 0.03, *p* = 0.89; wellness group: r = 0.01, *p* = 0.97), time since diagnosis (I-TOPS: r = 0.31, *p* = 0.17; wellness group: r = −0.24, *p* = 0.31) and SES (I-TOPS: r = −0.07, *p* = 0.77; wellness group: r = −0.10, *p* = 0.68).

The following demographic and clinical variables did not show a correlation with training satisfaction: FSIQ (I-TOPS: r = −0.01, *p* = 0.97; wellness group: r = 0.02, *p* = 0.94), time since diagnosis (I-TOPS: r = −0.31, *p* = 0.17; wellness group: r = 0.22, *p* = 0.34) and SES (I-TOPS: r = 0.09, *p* = 0.70; wellness group: r = 0.13, *p* = 0.58).

### Preliminary data on training efficacy

CBCL 6–18 total score [74] improved from T0 to T1 in the I-TOPS-group (Wilcoxon T(19) = 8.50; *p* < 0.01) but not in the wellness group (Wilcoxon T(15) = 44.00); *p* = 0.29), suggesting I-TOPS efficacy in improving participants’ adjustment. Whole data on training efficacy will be published in a subsequent paper. Intention-to-treat analyses will be used, thus all adolescents randomized to intervention groups will be considered in the analyses, including drop-outs. This will ensure an adequately powered final sample for endpoint evaluation.

### Safety and Adverse Events

Neither group reported any safety issues or adverse events, indicating that both the I-TOPS and the wellness interventions were well tolerated.

## Discussion

This study examined the feasibility of the Italian version of the TOPS program (I-TOPS), designed to address everyday EF in adolescents with ABI, in its first implementation within the Italian context and in comparison with an active wellness control intervention. Both interventions were delivered through a web-based platform with identical graphics and technical features, and both included remote supervision by a psychologist, who conducted online meetings at the end of each platform session.

To date, no remotely delivered program targeting everyday EF has been implemented in Italy for pediatric ABI or other neurological conditions [63,64,83]. Moreover, this is the first study offering TOPS to non-English-speaking participants after cultural and linguistic adaptation of the web-based platform [39]. No non-English-speaking European country has previously implemented TOPS: a feasibility RCT was conducted in Great Britain, but no data have been reported [84] and although a Spanish version exists, it has only been used in the USA with Spanish-speaking families [57]. In addition to evaluating the feasibility of implementing the intervention itself, we also examined the feasibility of the study design and procedures to determine whether the methodology was sustainable and replicable. To ensure comparability with previous research on remote cognitive training for pediatric patients with ABI, we applied a set of 9 feasibility criteria that have been used in earlier investigations [66,67].

Willingness to participate in the study was modest (61.73%) and below prespecified targets, although comparable to or higher than rates reported in American TOPS studies [55,56,58]. Several factors may have contributed to refusal, including stigma associated with psychological treatment, discomfort with technology, limited perceived need and competing family demands [57]. These barriers are consistent with prior literature on mental health stigma in brain injury populations, which highlights concerns about the usefulness of services, limited trust in providers, fear of others’ perceptions and feelings of discomfort. Additional reasons for declining participation may reflect family-level barriers previously identified in TOPS research, such as reluctance to acknowledge ABI-related difficulties, prioritization of other activities or high levels of family conflict [57,85].

A smaller proportion of families (12.90%) declined due to discomfort with technology. Notably, the online delivery modality appeared to be less of a barrier than other factors, aligning with evidence from the American context showing that online treatments are often perceived as an attractive alternative to clinic-based interventions for adolescents with ABI [55]. In one study examining treatment delivery preferences, adolescents and their parents viewed a self-guided, family-based problem-solving program as more convenient and beneficial than a face-to-face intervention [55]. Preference for online formats may reflect the reduction of structural barriers to care, particularly distance from providers. Supporting this interpretation, a previous study conducted by our group on remote drill-based cognitive training for adolescents with ABI reported that more than three-quarters of contacted families agreed to participate [66], suggesting that refusal in the present study is unlikely to be primarily driven by technology-based rehabilitation.

Regardless of the specific barrier, the fact that nearly 38.00% of families declined participation suggests a potential selection bias. Families who enrolled may have been more motivated to address ABI-related issues, which could influence scalability and generalizability. This consideration will be particularly important when interpreting efficacy outcomes in the future study evaluating the effectiveness of I-TOPS. Several strategies may help increase willingness to participate in future trials. Presenting I-TOPS within stakeholder organizations (e.g., brain injury support groups or rehabilitation centers) may help reduce barriers related to mental health stigma, reluctance to acknowledge ABI-related difficulties, prioritization of other activities or high family conflict, as suggested in previous TOPS research [57]. Positioning the program as complementary to existing services and strengthening education and collaboration with professionals across different subspecialties could further support this goal [57]. To address technology-related barriers, providers could organize informational sessions for patients and families to increase awareness of the intervention and foster a sense of connection to it. These sessions may also help families understand how to navigate challenges associated with using a web-based platform. When feasible, providers could preview selected web-based program sessions in person and demonstrate to parents and adolescents some of the skills they would acquire through the intervention [57]. These strategies directly address the barriers identified and may enhance scalability in real-world settings.

Provision of a remote cognitive intervention delivered through a web-based platform proved to be a viable rehabilitation solution for adolescents with ABI, supporting training accessibility. Adolescents in both groups were able to understand the program content and complete the proposed activities. Previous research has shown that high treatment complexity and limited treatment knowledge can hinder compliance [86] and in this study the pre-treatment meetings with the psychologist, during which families received instructions on accessing the website and connecting to Google Meet for videocalls, likely supported adherence and helped build rapport.

Technical smoothness was also high. No family experienced significant difficulties in accessing or completing the online modules, indicating that technological solutions can be successfully adopted by this population. Only two participants encountered minor issues (lost login credentials or video playback problems), all of which were promptly resolved after contacting the help center, an important component for ensuring the feasibility of online-based training. Overall, these findings suggest that technology-based interventions can be reliably delivered even in contexts, such as Italy, where digital readiness might be perceived as a barrier [87].

Among families who agreed to participate, the criteria for training adherence and training satisfaction were met only in the I-TOPS group. All participants assigned to this group completed baseline assessments, initiated the training, and only 2 dropped out, whereas the wellness group showed a three-fold higher dropout rate (6 dropouts). Satisfaction data help clarify this pattern: 84.21% of I-TOPS participants reported positive satisfaction with the intervention, compared with 60.00% in the wellness group. Higher satisfaction in I-TOPS was linked to perceived greater usefulness and enjoyableness of the program (items 8, 11, 13, and 14), as well as to a subjective perception of improved ability to handle crises (item 3).

The observed differences between groups may be explained by the inclusion of problem-solving content within the I-TOPS intervention, an element not incorporated into the wellness intervention. This component likely supported participants in managing everyday challenges, thereby increasing perceived usefulness [85,88,89]. These findings suggest that lengthy remote interventions are feasible when perceived as relevant and engaging. Importantly, in the wellness group, dropout rates were higher and satisfaction lower among older adolescents. This pattern may reflect their greater access to health and wellness information from other sources, which could have contributed to lower satisfaction; however, this hypothesis requires further validation. Conversely, the absence of an age effect on training satisfaction and adherence in the I-TOPS group suggests that the program’s content holds broader relevance across the adolescent age range.

Twice-monthly videocalls with the psychologist in the I-TOPS group may have further promoted engagement, given their focus on implementing lessons from the program into daily life. The benefits of teletherapy have been widely documented, including improved progress monitoring, personalized feedback and the use of motivational interviewing to sustain compliance [90–93]. Although the wellness group also received regular videocalls, these were briefer and focused primarily on reviewing program content and promoting adherence, without directly addressing adolescent or family difficulties through problem-solving, which may have limited their motivational impact compared with I-TOPS.

In sum, the findings suggest that remote interventions perceived as useful and enjoyable may be feasible for adolescents with ABI and their families. Overall perceptions of I-TOPS helpfulness were consistent with prior studies [55,88–90], indicating that the Italian adaptation of the program was also viewed as convenient and beneficial.

In our sample, 21.05% of adolescents of the I-TOPS and 20.00% of the wellness group who concluded the training (completers) showed low intellectual functioning (FSIQ: 70–79) and they all succeeded in performing the program and the related assessment. Metacognitive abilities are required to perform the I-TOPS program and one may speculate that adolescents with higher intellectual proficiency would have higher adherence rates or greater satisfaction. However, a previous meta-analysis [94] indicated that adolescents with lower FSIQ may be the most likely to benefit from online problem-solving treatment;. Up to now, no data on the relation between FSIQ and training feasibility are available for the American context. In our sample, FSIQ was not correlated with completion rates or satisfaction questionnaire scores, suggesting that training feasibility and satisfaction did not vary across different levels of cognitive ability. Furthermore, unlike previous research conducted in the USA [55], which found that lower SES was associated with more positive program evaluations, we found no such correlation. This discrepancy may reflect the Italian context: on average, more than 5 years after diagnosis (study mean time since the ABI), families often face a shortage of services for adolescents, particularly those integrating cognitive and mental health support. More broadly, the shortage of professionals trained in specialized rehabilitation and the limited availability of standardized treatment protocols for neurological disorders in Italy appear to affect families across all socioeconomic levels. As a result, even families with higher SES, who would typically have greater access to resources, may find their ability to benefit from available local services limited and therefore may provide similarly positive evaluations of a remote support intervention as families with lower SES.

Our findings regarding the feasibility of assessment timescale and procedures raise important considerations regarding study methodology. The collection of outcome measures within the targeted timeframe was feasible for the majority of families. Specifically, 94.74% of families in the I-TOPS group and 86.67% in the wellness intervention group completed post-training assessments according to the pre-specified research schedule, while 88.89% of families in the I-TOPS group and 92.31% in the wellness intervention group did so at the 6-month follow-up. However, the predefined criterion was not fully met in either group, as it was expected that 100.00% of families would complete assessments within the established timelines. These findings suggest the need to increase flexibility in the assessment schedule for future studies or to contact families earlier, allowing sufficient time to reschedule assessments around personal commitments. In the present study, families were contacted one week before the planned assessment as a reminder to attend the appointment and complete the questionnaires, which may have been insufficient notice. Additionally, a reduction in the number of required questionnaires should be considered, as this study included 4 questionnaire-based measures for adolescent outcomes and 3 for parent outcomes.

Furthermore, an issue emerged regarding the collection of questionnaires on parent functioning, as some parents explicitly refused to complete them even at the baseline assessment. Specifically, they felt that reporting on their psychological well-being was not relevant. While this issue did not arise in the American context, it is possible that, in Italy, concerns about privacy and stigma associated with mental health difficulties persist, discouraging parents from providing information about their psychological functioning [95,96]. This represents an important consideration when designing research that examines the functioning of relatives of patients receiving rehabilitation interventions, as such reluctance may undermine data collection or bias findings. Parents’ potential discomfort with completing measures about their own functioning has also been noted in previous research [97]. In a study involving parents of children with epilepsy, only half of the approached families agreed to participate, and among those who consented, several returned the questionnaires unanswered. In the present study, 28.57% of parents in both groups at baseline chose not to return questionnaires on their psychological well-being [97]. As a result, data on intervention effects for parents will need to be interpreted with caution due to possible reporting bias and limited generalizability. In future research, this barrier could be reduced by providing clearer explanations of the purpose of parent questionnaires and by adopting more neutrally worded formats that focus on general aspects such as stress or family routines rather than explicitly on ‘mental health’ or ‘psychological problems’. Such adjustments may help parents feel less judged and more comfortable completing the measures.

Finally, loss to follow-up did not pose an issue at the immediate post-treatment assessment, as all adolescents and families who completed the interventions also completed the first follow-up evaluation. At the 6-month post-training follow-up, loss to follow-up rates were 5.30% in the I-TOPS group and 13.30% in the wellness group, meeting the predefined criterion but suggesting a potential risk of underpowered statistical analyses in studies with a longer interval between intervention completion and follow-up. Because evaluations scheduled many months or even years after treatment are crucial for assessing the maintenance of treatment gains, it would be useful to report statistical power at each assessment point or to adopt statistical methods that account for dropouts in efficacy analyses, as planned for future evaluations on efficacy in the present study.

Limitations of this study should be acknowledged. First, even though the research design and procedures used for this RCT proved to be a valid method for conducting investigations on the topic, the presence of a high percentage of families who did not agree to participate in the study indicates the existence of a potential selection bias. This may compromise generalization, since the families who joined the project may also have been the most motivated to receive the training and, therefore, may have responded more positively to outcome measures. Second, a warning has been raised about the possibility of collecting and subsequently interpreting data on parents. In fact, about 20.00% of parents in each study group refused to complete questionnaires on their own functioning, indicating that data on the study’s effectiveness on parental functioning should be interpreted with caution. They might be influenced by a bias associated with the fact that parents with more problems might find it important to provide information about themselves or, conversely, might refuse to do so. This aspect could further contribute to limiting the generalizability of the results. Third, the primary focus of this feasibility study was on the usability of the program from the patients’ perspective and on the feasibility of the study procedures within the target population. No direct attention was given to the therapists involved in delivering the interventions. However, future studies could broaden the scope to include clinicians’ perspectives, using methods such as semi-structured interviews or focus group discussions, to obtain a more comprehensive understanding of the intervention’s feasibility and its potential for integration into clinical practice. Fourth, this study focused on adolescents in the chronic phase following ABI; therefore, the results may not be generalizable to those in acute care settings or undergoing subacute rehabilitation at specialized centers. This limitation also relates to the content and focus of the intervention, which was specifically developed to address the needs of patients in real-life ecological settings. Finally, although the study was double-blind, participants might still have inferred their group assignment based on intervention content, even if group labels were not disclosed. While the interventions were comparable in terms of platform graphics, duration and timing of assessments and therapist supervision, which may have reduced this issue, future studies could incorporate blinding checks to quantify and mitigate this potential bias.

## Conclusions

The use of technological solutions for the rehabilitation of adolescents with ABI appears to be an acceptable and feasible mode of intervention, perceived as convenient and beneficial by both adolescents and their families. The present study demonstrated the feasibility of the I-TOPS intervention, with participants showing high adherence and satisfaction compared with a wellness active control. Intervention fidelity was maintained throughout the study, with all core components of I-TOPS delivered as intended and supported by regular clinical supervision. No safety concerns or adverse events were reported, indicating that the intervention can be delivered safely in a remote format.

Taken together, the feasibility measures from this study point to a consistent picture. The main difficulty lies in the initial uptake of the intervention, likely influenced by stigma, competing family demands and some degree of technological discomfort. Once enrolled, however, families in the I-TOPS group showed strong engagement and positive satisfaction with the program. Technological feasibility was excellent, with only minimal issues reported and adequate comprehension of the activities to be completed on the web-based platform, indicating that the digital format of the intervention is unlikely to hinder participation. The high levels of therapeutic alliance and satisfaction observed in the I-TOPS group were not restricted to affluent or high-functioning families and did not vary by age or FSIQ range, suggesting broad relevance across diverse adolescent and family profiles.

In contrast, challenges emerged with study procedures and the generalizability of the findings. Approximately 38.00% of contacted families declined participation, limiting applicability to those willing to engage in a family-based cognitive and psychological intervention. Participation in future trials may be improved by presenting I-TOPS within stakeholder organizations and strengthening collaboration with professionals to reduce stigma-related and motivational barriers. Brief informational sessions to address technological concerns could further increase family confidence and connection to the program, supporting its broader scalability. Additional difficulties were observed in respecting assessment scheduling by families and in the collection of parent-reported measures, which will require refinement to improve response rates. Allowing families more time to complete questionnaires and adopting shorter, more neutrally worded measures focused on general domains such as stress or family functioning, rather than psychological functioning, may help reduce these barriers. Providing assessment reminders earlier than one week in advance may also support families in organizing themselves to attend scheduled appointments. Aside from these issues, loss to follow-up and overall participation rates were acceptable, demonstrating that once families agree to participate, sustained engagement is achievable.

Overall, the results support the viability of progressing toward larger studies, provided that targeted strategies are implemented to address the identified issues and enhance scalability. Finally, the study required modest technological and personnel resources, suggesting that implementation at scale is feasible.

## Supporting information

S1 Protocol Trial protocol as approved by the Ethic Committee. (DOCX)

S1 Checklist CONSORT 2010 checklist. (DOC)

S1 Data Original database. (XLSX)
