# Supplementary material for: Feasibility of the Italian version of the Teen Online Problem Solving Program (I-TOPS) in a sample of adolescents with acquired brain injury: Results from a randomized controlled trial
Source: PLoS One. 2026 Jul 28;21(7):e0354280. doi: 10.1371/journal.pone.0354280 (PMC13411921; doi:10.1371/journal.pone.0354280)
Supplement: S1 Protocol — (DOCX) [file pone.0354280.s001.docx]

| **ID and version:** | Id. **843-** version no.**0** of 23.12.2020 |
| --- | --- |
| **TITLE of the PROJECT:** | Rehabilitation of executive functions and social skills in adolescents with neurological condition: use of the Italian version of the TOPS platform (I-TOPS) |
| **SCIENTIFIC MANAGER:** | Dr. Alessandra BARDONI |
| **LEAD/PROMOTER:** | IRCCS E. MEDEA – Scientific Area of Associazione “La Nostra Famiglia”, Bosisio Parini (LC) |
| **ENTITIES INVOLVED:** | / |
| **RESEARCH AREA: The European Commission** | Clinical neuroscience of the evolutionary age in the field of rehabilitation (neuropathology, neurophysiopathology and rehabilitation) |
| **FUNDING:** | 5 per 1000 funds for biomedical research |
| **REFERENCE CALL:** | / |
| **DURATION: The** | 24 MONTHS |
| **STUDY TYPE:** | INTERVENTIONAL STUDY |

**ABSTRACT**

This study is part of the Ricerca Corrente project titled "Specific rehabilitation treatments in pediatric patients with severe acquired brain injury: from vegetative state to functional recovery", coordinated by Dr. Sandra Strazzer, who serves as the project administrator.

The aim of the present study is to evaluate the feasibility and efficacy of the Italian version of the Teen Online Problem-Solving intervention (TOPS) in patients with neurological conditions (e.g., non-progressive acquired brain injury, brain tumor, epilepsy, etc.). This clinical population often exhibits difficulties in executive functioning and social skills, and the TOPS program, originally developed in the United States by Dr. Shari Wade, was specifically designed to improve these domains.

The implementation of an innovative home-based intervention for children with neurological conditions represents a milestone in the provision of remote treatments targeting everyday functional abilities and the quality of life of both patients and their families. To date, no remote interventions are available in Italy that specifically address the improvement of executive functioning and behavioral issues in children with neurological conditions, despite a high demand for such support. Based on the existing literature demonstrating the efficacy of the original TOPS program, we hypothesize that the Italian version (I-TOPS) will also be feasible and effective within the Italian clinical population. The present study adopts an experimental research design to compare two groups: an experimental group undergoing the I-TOPS program and a control group participating in a web-based program that mirrors I-TOPS in structure but lacks the content specifically aimed at enhancing executive functioning and social skills.

Fammi sapere se desideri una versione più sintetica, più tecnica, o tradotta in italiano.

**METHODOLOGICAL ASPECTS**

**Background**: Everyday executive dysfunctions, along with behavioral and social difficulties, are common in adolescents with neurological conditions. Targeted interventions addressing these issues can significantly enhance patients’ quality of life.

In this context, the Italian version of the Teen Online Problem-Solving intervention (I-TOPS) appears to be a suitable rehabilitation tool. The intervention consists of a computerized platform that provides psychoeducational content and instructional videos on problem-solving strategies. These materials are designed to be accessed independently at home by patients and their families. In addition, psychological support is offered to facilitate the application of these strategies in real-life situations, helping adolescents manage challenges related to executive functioning, behavior, and social interactions.

The original TOPS program, developed and initially implemented in the United States, has been evaluated in numerous studies. Clinical trials and subsequent meta-analyses have demonstrated its feasibility and efficacy, showing positive outcomes for both adolescents and their families.

**Main objective:** the main aim of the study is to evaluate the feasibility of the I-TOPS program and its efficacy in improving cognitive/behavioral functions of adolescents with neurological condition.

**Secondary objective:** the secondary objective is to assess the impact of the program on the psychological functioning of parents. As a family-centered approach, it is expected that the I-TOPS program also generates benefits in the parents of participants, as indicated by international scientific literature.

**Notes on the calculation of sample size:** A final sample of 42 adolescents will be included, as determined by power analysis. This sample size will allow a preliminary assessment of the feasibility and efficacy of the program. Based on randomization, half of participants will be allocated to the I-TOPS program and the other half to an active control program (wellness intervention). The control intervention, although presenting the same structure as the I-TOPS intervention, will not address everyday executive functions and social skills.

**Activities planned:** After the approval of the Ethic Committee, enrollment will start. Specifically, parents of eligible children will be contacted by the referring physician and receive detailed information about the study and its objectives. If they accept participation, they will be asked to sign the informed consent.

Adolescents will then be randomized into study groups (randomization will be performed by an independent statistician using a dedicated software).

Patients in the experimental group will be offered the I-TOPS program, while those in the control group will be offered the wellness intervention, that will include health and wellness content without addressing everyday executive functions. With respect to the I-TOPS program, prior to training start, a psychologist with expertise in cognitive-behavioral therapy will organize a meeting with the adolescents and families aimed at defining everyday goals to be achieved through the training. After this initial assessment phase, adolescents and families will be asked to complete the activities in the I-TOPS sessions, in accordance with a predefined timeline of 2 weeks per each session.

At the end of each session, a Google Meet video call will be held with the psychologist to discuss the content learned and the exercises completed and, if necessary, review the strategies used to achieve the daily life goals defined in the assessment phase. For the control group, there will be no pre-training assessment on the objectives to be achieved through the program; further, contact with the therapist will have the exclusive aim of sustaining the motivation to carry out the intervention, not of working on everyday skills.

The total duration of the intervention (both for the experimental and control group) will be 6 months.

The computer-based platforms of both programs will enable clinicians and researchers to monitor sessions and exercises performed. At the conclusion of the interventions, questionnaires on the feasibility and accessibility of the training will be administered to both adolescents and parents.

In addition, clinical assessments of patients’ and parents’ functioning will be conducted before the intervention (T0), immediately after the intervention (T1) and at a 6-month follow-up (T2). Specifically, changes in adolescents’ everyday executive functions as well as their psychological and behavioral functioning will be evaluated, while changes in parents’ psychological well-being will also be assessed.

The following assessment tools will be used:

- CBCL 6-18 (Questionnaire filled by parents on the functioning of adolescents);
- YSR 11-18 (Questionnaire filled by the adolescents on their own functioning);
- BRIEF-II Parent Form (Questionnaire filled by parents on the functioning of adolescents);
- BRIEF-II Self Report Form (Questionnaire filled by the adolescents on their own functioning);
- Symptom Checklist-90-R (Questionnaire filled by parents on their own functioning);
- Back Anxiety Inventory (Questionnaire filled by parents on their own functioning);
- Parenting Stress Index (Questionnaire filled by parents on their own functioning);
- Jansari assessment of Executive Functions (JEF): a computer-based evaluation of everyday executive functions to be completed by adolescents;
- NEPSY-II Affect Recognition and Theory of Mind subtests: these subtests assess social cognition and will be administered to adolescents.

All data collected for the study will be entered into an encrypted database (with password access), stored on a computer protected by password at IRCCS E. Medea, Bosisio Parini, Lecco. Any direct reference to the patient’s identity will be deleted from the database.

Following post-treatment evaluations, feasibility results will be examined using 9 criteria taken from extant literature; to evaluate training efficacy, intention-to-treat analyses will be performed.

**Expected results:**

- **I-TOPS program is feasible for patients (limited drop-outs, good satisfaction rates, no significant technical issues etc.);**
- **positive rates on I-TOPS satisfaction by adolescents and parents;**
- **improvement of everyday executive functions and behavioral/psychological functioning in adolescents receiving I-TOPS;**
- improvement of psychological functioning in parents of adolescents receiving I-TOPS;
- **improvement of psychological well-being of parents of the control group, due to a potential improvement of their children’s lifestyle after the wellness intervention. However, such improvement is expected to be lower than the one expected for the I-TOPS group.**

**DESCRIPTION OF THE POPULATION**

**Characteristics of subjects/patients: adolescents (11-19 years) with neurological condition (non-progressive acquired brain injury, brain tumor, epilepsy etc.) and without photosensitive epilepsy will be involved.**

**Total subjects/patients N.** 42 adolescents

**No. subjects/patients per center:** 42 adolescents

**Justification of sample size: a sample of 42 patients per neurological condition will be involved, based on power analysis. This sample size will allow a preliminary assessment of the feasibility and efficacy of the I-TOPS program.**

**Inclusion criteria:**

- diagnosis of neurological condition (e.g., non-progressive acquired brain injury, brain tumor, epilepsy etc.);
- age between 11 and 19 at recruitment;
- proper comprehension and speaking abilities in the Italian language;
- parents or legal guardians available to participate in the intervention.

**Exclusion criteria**:

- other rehabilitation interventions on cognitive/behavioral functions;
- presence of preinjury or comorbid conditions;
- photosensitive epilepsy.

**General criteria for evaluating effectiveness:**

**The following tools will be used for assessing effectiveness:**

- CBCL 6-18 (Questionnaire filled by parents on the functioning of adolescents);
- YSR 11-18 (Questionnaire filled by the adolescents on their own functioning);
- BRIEF-II Parent Form (Questionnaire filled by parents on the functioning of adolescents);
- BRIEF-II Self Report Form (Questionnaire filled by the adolescents on their own functioning);
- Symptom Checklist-90-R (Questionnaire filled by parents on their own functioning);
- Back Anxiety Inventory (Questionnaire filled by parents on their own functioning);
- Parenting Stress Index (Questionnaire filled by parents on their own functioning);
- Jansari assessment of Executive Functions (JEF): a computer-based evaluation of everyday executive functions to be completed by adolescents;
- NEPSY-II Affect Recognition and Theory of Mind subtests: these subtests assess social cognition and will be administered to adolescents.

**General criteria for the evaluation of acceptability: the evaluation of acceptability and feasibility of the I-TOPS and wellness intervention will be carried out through the administration of ad-hoc questionnaires to both adolescents and parents. The feasibility of also the study procedures will be assessed.**

**Specifically, the feasibility of the training and study procedures will be analyzed using the following measures:**

- **adherence to the study;**
- **adherence to the training;**
- **number of drop-outs;**
- **number of sessions completed in the target time;**
- **technical problems encountered;**
- **data obtained from a questionnaire on training satisfaction;**
- **data on assessment procedures and patients’ retention rates at assessment points;**
- **participation willingness.**

**Statistical methodology: indicate % of expected drop-outs:** descriptive analyses of the demographic and clinical characteristics of the sample will be conducted. Inferential analyses on pre- versus post-treatment differences in the overall group will be performed using an intention-to-treat approach.

**Expected drop-out: 10%.**

**RISK/BENEFIT ASSESSMENT**

**Possible benefits:**

**The benefits of the I-TOPS program can be summarized as follows:**

-possibility of implementing an innovative home-based intervention targeting cognitive skills and behaviors that significantly impact the everyday functioning of adolescents with neurological conditions (an intervention with ecological validity);

-**p**ossibility of providing rehabilitation access to patients living far from specialized centers**;**

**-p**ossibility of delivering a family-centered intervention that directly involves parents in the rehabilitation process of their children, with associated benefits supported by scientific literature**;**

**-**sharing the clinical practice methodology used in relation to the I-TOPS program at both national and international levels, through dissemination of results via scientific publications and presentations at conferences and congresses.

**Possible disadvantages and risks:** the risks associated with this project are very low, as interventions are delivered under constant family supervision and biweekly clinician oversight. Furthermore, the interventions do not involve content related to psychological trauma, thereby minimizing the risk of triggering strong emotional distress in the absence of a clinician. One potential risk of the proposed interventions could be prolonged computer use; however, this will be mitigated by excluding patients with photosensitive epilepsy.

**Diagnostic and therapeutic alternatives:** a possible therapeutic alternative is the delivery of interventions on everyday executive functions or health and wellness content in the clinical setting.

**Study procedures:**

-patient recruitment and completion of informed consent;

-delivery of the allocated remote intervention;

-collection of outcome measures before and after treatment;

-analysis of the feasibility and efficacy of I-TOPS compared to the wellness intervention;

-dissemination of results through conferences, congresses and scientific publications.

**Precautionary measures to safeguard subjects/patients: due to the prolonged use of an electronic device, it is planned to exclude patients with photosensitive epilepsy.**

**Overall assessment of the risk/benefit ratio**: overall, it is estimated that the benefits related to the use of the rehabilitation programs indicated, in terms of potential benefits on patients’ functioning and family well-being and reduction of the costs of interventions, are significantly higher than the possible risks associated with the use of electronic devices for long time periods. Furthermore, the exclusion of patients with photosensitive epilepsy should limit the risks associated with the prolonged use of an electronic device.
